# Effects of Probiotic Supplementation on Core Symptoms of Autism Spectrum Disorder in Children

**DOI:** 10.3390/nu18071127

**Published:** 2026-03-31

**Authors:** Meng Xian Chan, Chui Yan Hoh, Ying Yi Teh, Xin Yian Toh, Noor Akmal Shareela Ismail

**Affiliations:** Department of Biochemistry, Faculty of Medicine, Universiti Kebangsaan Malaysia, Jalan Yaacob Latif, Bandar Tun Razak, Kuala Lumpur 56000, Malaysia; a188342@siswa.ukm.edu.my (M.X.C.); a190102@siswa.ukm.edu.my (C.Y.H.); a188721@siswa.ukm.edu.my (Y.Y.T.); a188077@siswa.ukm.edu.my (X.Y.T.)

**Keywords:** gut–brain axis, gut microbiota, *Lactobacillus reuteri*, psychobiotics, pediatric, autism, microbiome-gut-brain axis

## Abstract

**Background/Objectives**: Increasing evidence implicates the microbiome–gut–brain axis in Autism Spectrum Disorder (ASD) pathophysiology, prompting interest in probiotics as a therapeutic strategy, although findings remain inconsistent. This systematic review aimed to evaluate the clinical efficacy of probiotic supplementation on core ASD symptoms, examine the outcome measures used, and provide insights into optimal probiotic interventions. **Methods**: This review was conducted in accordance with PRISMA 2020 guidelines. PubMed, Scopus, Web of Science, Ovid, ProQuest, and Wiley Online Library were searched for studies published between January 2015 and July 2025. Randomized, non-randomized, and open-label clinical studies evaluating oral probiotic supplementation in children and adolescents with ASD were included. Outcomes assessed core symptom domains using validated instruments. Study selection, data extraction, and risk-of-bias assessment (RoB 2 and ROBINS-I) were performed independently by multiple reviewers. Due to methodological heterogeneity, the findings were synthesized narratively. **Results**: Fourteen studies involving 924 children and adolescents with ASD across seven countries or regions were included, of which ten were randomized controlled trials. Eight studies reported significant improvement in core ASD symptoms, predominantly within the social and communication domain. The most frequently used assessment tools were the Social Responsiveness Scale (SRS), Autism Treatment Evaluation Checklist (ATEC), and Autism Diagnostic Observation Schedule (ADOS). *Lactobacillus reuteri* supplementation for at least three months was consistently associated with improvement in social behavior. **Conclusions**: *L. reuteri* supplementation possibly improves social and communication function in children with ASD. However, there is limited high-quality evidence on this. Evidence for other core domains remains limited and inconsistent, highlighting the need for well-designed, multicenter trials using standardized outcome measures and strain-specific hypotheses.

## 1. Introduction

Autism Spectrum Disorder (ASD) is defined in DSM-5 by persistent deficits in social communication and social interaction, as well as restricted/repetitive behavior patterns [1]. Recent years have seen a dramatic rise in ASD prevalence: from 4 in 10,000 individuals in 1978 to 1 in 100 children diagnosed worldwide by 2022 [2,3,4,5]. This increase can be attributed to expanded diagnostic criteria, improved screening and services, diagnostic substitution, changing study methodologies, and rising risk factors [6,7]. As a result, the growing prevalence demands additional healthcare resources for psychotherapy, autism-friendly environments, NCD prevention, and novel treatments [8,9,10,11].

Various tools are available to assess symptom severity of ASD, such as the Autism Diagnostic Observation Schedule—Second Edition (ADOS-2) [12], the Childhood Autism Rating Scale—Second Edition (CARS-2) [13], the Autism Behavior Checklist (ABC) [14], the Autism Treatment Evaluation Checklist (ATEC) [15], the Vineland Adaptive Behavior Scales, Second Edition (VABS-II) [16], and the Social Responsiveness Scale [17]. The variety of tests is due to differences in test settings and purposes. Some rely on parent or carer reports, and others use observation and interview. Many of these tests are used to standardize aspects of history-taking and physical examination; others are used to shorten diagnostic interviews and reduce costs, especially in research studies [18]. A recent meta-analysis found that, among the tools for diagnosing ASD, CARS and ADOS had the highest sensitivities, at 89% and 87%, respectively, with specificities of 79% and 75%, respectively [19]. Apart from CARS and ADOS, other tools also demonstrated good validity in assessing ASD severity [20,21,22,23]. The tools mentioned above measured four core domains: “Social & Communication”, “Restrictive & Repetitive Behavior”, “Sensory & Cognitive Processing”, and “Adaptive Functioning”.

Over the past decade, evidence of the microbiome axis has shown immense potential as a novel treatment target for ASD and other psychiatric and neurological disorders [24,25]. The microbiome axis includes the central nervous system, the autonomic nervous system, the enteric nervous system, the enteric microbiota, and the hypothalamic–pituitary–adrenal axis [26]. Researchers found that enteric microbiota are responsible for converting precursors to neurotransmitters and producing neuroprotective metabolites, which, in turn, may influence human emotions by regulating the gut–brain axis [27]. Strong and clear separation had been found between the gut microbiome profiles of children with ASD and their age- and sex-matched neurotypical peers, further consolidating the association between the gut–brain axis and ASD [27,28]. Thus, researchers began experimenting with microbiome therapeutics, including probiotics and fecal microbial transplantation (FMT), in ASD patients to introduce beneficial microbes into the gut microbiota [27]. The first clinical trial using probiotics as an intervention to observe the effect on the core symptoms and gastrointestinal symptoms in children with ASD was conducted in 2019 [29], and subsequent studies on similar topics have been published to date [30,31].

Not only is the difference in gut microbiota found between neurotypical individuals and individuals with ASD, but the gut–brain axis may also be a key determinant factor in the severity of ASD symptoms. *Desulfovibrio* spp. was found to have a strong association with the severity of autism [32], and another study found that gastrointestinal symptom severity was strongly correlated with ASD severity, as assessed by the ATEC score, suggesting the underlying pathophysiology of the gut–brain axis in affecting the severity of ASD symptoms [33].

ASD is a heterogeneous disorder with a variable presentation, including Autistic Disorder (AD), Asperger Syndrome (AS), and, prior to the 2013 edition of DSM-5, Pervasive Developmental Disorder Not Otherwise Specified (PDD-NOS) [1]. Is there evidence of microbiota dysbiosis present in the heterogeneous presentation of ASD, which would justify the use of probiotic intervention across the spectrum in ASD? A recent study revealed that AS children in a prospective follow-up design had reduced levels of Bifidobacterium, Bacteroides, Lactobacillus, Enterococcus, and other microbes compared to neurotypical controls, proving that gut dysbiosis is present in AS [34]. A case reporting the application of FMT in adult AS showed a change in the structure of intestinal microbiota with the amelioration of symptoms of AS, including gastrointestinal symptoms [35]. These findings suggest that, AS being the second most common presentation within ASD after AD, individuals with AS may similarly benefit from probiotic intervention and other microbiota therapy. Unfortunately, there were no trials of gut microbiome analysis and microbiota therapy in the PDD-NOS cohort. Future studies with more comprehensive and accurate phenotyping of ASD presentation are needed.

Despite the increasing evidence on the gut–brain axis and increasing clinical trials on probiotic supplementation in ASD, the findings remain inconsistent. Some studies have reported improvements in gastrointestinal symptoms, behavior, or sleep quality, whereas others found minimal or no therapeutic effect [30,36,37]. These discrepancies may be attributed to variations in study design, probiotic strains, treatment duration, outcome measures, and small sample sizes.

The primary research gaps are the lack of standardization in interventions and outcome measures. A wide variety of probiotic formulations, such as single strains, multi-strain mixtures, and combinations with prebiotics or other dietary components, were used across different studies. Nowadays, it is widely explored using mixtures of strains as supplements in clinical trials for children with Autism Spectrum Disorder (ASD) [30,38]. The effects of probiotics are known to be strain-specific. However, most of the trials do not test individual strains within their mixtures, leaving a gap in understanding which specific bacterial strains are responsible for the observed benefits [37]. There are various assessment tools used to measure outcomes, making it challenging to synthesize findings across studies in a systematic review. In addition, confounding factors such as dietary habits, which significantly influence the gut microbiota, are often not standardized or adequately controlled [31,38]. Similarly, many children with ASD receive concurrent behavioral therapies, which can confound the results and contribute to improvements seen in both placebo and treatment groups [30,36].

Given the lack of standardization in clinical trials, the efficacy of probiotic interventions on core ASD symptoms remains inconclusive. Therefore, this study aimed to review the clinical efficacy of probiotic supplementation on core ASD symptoms and to identify responsive domains (social and communication, restrictive and repetitive behavior, sensory and cognitive processing, and adaptive functioning) in pediatric ASD patients. This study also aimed to review the evaluation tools used to assess the severity of core ASD symptoms among studies of probiotic supplementation. The findings were expected to provide insights into the potential of the recommended probiotic intervention in patients diagnosed with ASD and to guide improvements in the design of studies using probiotic intervention.

## 2. Materials and Methods

**Figure 1:**
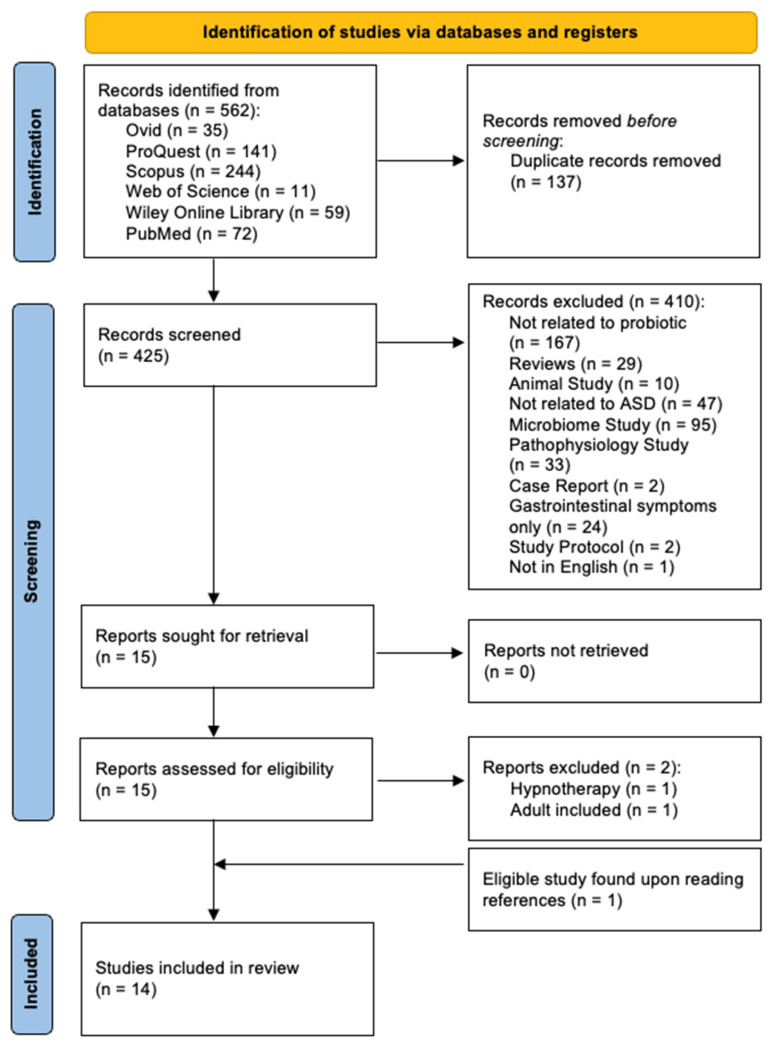
Flow diagram of studies evaluated in the systematic review based on the PRISMA 2020 statement.

This systematic review was conducted in accordance with the Preferred Reporting Items for Systematic Reviews and Meta-Analyses (PRISMA) 2020 [39] guidelines. A PRISMA 2020 flow diagram was used to illustrate the study identification, screening, and selection process (Figure 1).

The eligibility criteria were predefined using the Population–Intervention–Comparator–Outcome (PICO) framework. The population comprised children and adolescents aged 18 years or younger diagnosed with Autism Spectrum Disorder (ASD) based on established diagnostic criteria (DSM-IV, DSM-5, or ICD) or validated diagnostic instruments. The intervention of interest was oral probiotic supplementation, including single-strain or multi-strain formulations, with or without the addition of prebiotics (synbiotics). Comparators included placebo, standard care, alternative probiotic formulations, or no comparator in the case of non-randomized or open-label studies. The outcomes focused on changes in core ASD symptom domains, including social communication, restricted and repetitive behaviors, sensory and cognitive processing, and adaptive functioning, assessed using validated behavioral or clinical instruments. The eligible study designs included randomized controlled trials (RCTs), non-randomized controlled trials, and open-label clinical studies. Additional inclusion criteria were human studies, original research articles, English-language publications, and studies published between January 2015 and July 2025. The exclusion criteria comprised reviews, meta-analyses, protocols, case reports, conference abstracts, editorials, letters, animal or in vitro studies, fecal microbiota transplantation studies, non-probiotic interventions, and studies assessing gastrointestinal outcomes only without an evaluation of core ASD symptoms.

A comprehensive literature search was conducted in PubMed, Scopus, Web of Science, Ovid, ProQuest, and Wiley Online Library, covering publications from January 2015 to July 2025. Each source was last searched on 2 August 2025. The search strategy combined Medical Subject Headings (MeSH) and free-text terms related to probiotics, gut microbiota, and ASD using Boolean operators: (“probiotics” OR “psychobiotics” OR “microbiota” OR “microbiome” OR “Lactobacillus” OR “Bifidobacterium”) AND (“autism” OR “autism spectrum disorder” OR “Asperger syndrome”) AND (“children” OR “pediatric”). Reference lists of included studies and relevant reviews were manually screened to identify additional eligible articles.

All retrieved records were imported into a shared database, and duplicates were removed. Title and abstract screening were independently performed by four reviewers (MXC, CYH, YYT, and XYT), followed by a full-text assessment against the eligibility criteria. Disagreements were resolved through discussion, with a fifth reviewer (NASI) acting as adjudicator when consensus could not be reached. The study selection process is presented in a PRISMA 2020 flow diagram. A total of 562 records were initially identified through database searching. After the removal of duplicates, reviews, and non-research articles, 425 abstracts were screened. Studies were excluded if they were systematic reviews, case reports, meta-analyses, study protocols, dietary interventions, microbiome-only studies, fecal microbiota transplantation studies, or focused solely on gastrointestinal symptoms. Thirteen publications met the eligibility criteria, with one additional study identified through reference screening, resulting in a total of fourteen included studies.

Data extraction was performed independently using a standardized form and included the author and year, country, study design, participant characteristics, probiotic strain(s), dosage and duration, comparator, outcome measures for core ASD symptoms, main findings, study limitations, and evidence of attempts at controlling confounding factors. Discrepancies were resolved through consensus.

Methodological quality was assessed independently by two reviewers. Randomized controlled trials were evaluated using the Revised Cochrane Risk of Bias Tool (RoB 2) [40], assessing bias related to randomization, deviations from intended interventions, missing outcome data, outcome measurement, and selective reporting. Non-randomized studies were assessed using the ROBINS-I tool [41], evaluating bias due to confounding, participant selection, intervention classification, deviations from intended interventions, missing data, outcome measurement, and selective reporting. Studies were classified as having low, some concerns, serious, or critical risk of bias.

The results of individual studies were summarized in structured tables detailing study design, participant characteristics, probiotic interventions, outcome measures, main findings, and limitations. Additional tables were used to present findings according to ASD core symptom domains and to cross-tabulate reported improvements against risk-of-bias judgments. Risk-of-bias assessments were visually displayed using summary figures for randomized controlled trials and tabular formats for non-randomized studies.

Due to substantial heterogeneity in probiotic strains, dosages, intervention durations, outcome measures, and study designs, a meta-analysis was not performed. Specifically, marked variability was observed in probiotic strain composition, treatment duration, participant age ranges, baseline ASD severity, and outcome measurement tools. Although strain-restricted and duration-restricted subgroup analyses were considered during protocol development, these approaches were deemed infeasible because of the limited number of studies per probiotic strain and the wide variation in intervention duration categories, which would have resulted in underpowered and potentially misleading pooled estimates. Given these constraints, a narrative synthesis was conducted to preserve methodological rigor and avoid an inappropriate statistical aggregation of heterogeneous data. Findings were synthesized narratively and grouped according to ASD core symptom domains, probiotic formulation, intervention duration, participant age, presence of baseline gastrointestinal symptoms, study quality, and attempts to control confounding factors.

Robustness of findings was assessed qualitatively by examining the consistency of results across studies with different levels of risk of bias. Attention was paid to whether reported improvements were supported by studies with low or some concerns for risk of bias, and whether findings persisted when excluding non-randomized or high-risk studies. The certainty of evidence for each outcome domain was assessed using a structured qualitative approach considering risk of bias, inconsistency, indirectness, and imprecision.

This systematic review was not prospectively registered in PROSPERO, which is acknowledged as a methodological limitation. The review protocol was not prepared. GPT-5.2 was used to correct grammar errors during the writing of the manuscript.

## 3. Results

The data extracted from the 14 publications are summarized in Table 1.

### 3.1. Study Characteristics

Studies were conducted in seven different countries/regions: the U.S. (n = 2), Italy (n = 5), China (n = 2), India (n = 1), Taiwan (n = 2), Spain (n = 1), and Egypt (n = 1). Ten out of 14 studies are randomized controlled trials, reducing the sources of bias typical of studies measuring the efficacy of an intervention. The ASD subjects (n = 924) are predominantly males (577 males and 139 females; in three studies [29,36,38], the sex of the participants was missing); all are children and adolescents between 1.5 and 17 years old, with ASD described in studies published since 2018. The sample sizes are relatively small, ranging from 10 to 180 subjects.

Most studies (10 out of 14) [30,31,36,37,38,42,43,44,45,46] measured GI symptoms in their studies. Five studies performed gut microbiota or metabolite analysis [31,36,37,38,43]. Seven studies reported adverse events in the samples [29,36,37,42,43,44,47]. Five studies reported reasons for sample dropout [29,31,43,44,48]. The patients’ compliance with the intervention is only tracked in 4 studies (by telecommunication, follow-ups, or checking remaining doses [30,31,42,47]. Most of the studies excluded patients with a diagnosis of organic GI disorders, the usage of systemic antibiotics before and during enrollment, brain anomalies, neurological syndromes, special diets, usage of other probiotics, and known allergy to probiotics.

The type of intervention varies across the studies. The most common formulations used are the De Simone formulation (marketed as Vivomixx^®^ in Europe) (n = 3) and *Lactobacillus plantarum* PS128 (n = 3). Another study [31] combined dietary intervention with probiotics; one more study [36] combined ABA training with probiotics. The dosage of probiotics ranged from 0.4 to 100 billion CFUs/day, indicating a high variety in dosage selection among the studies. The duration of intervention varies from 4 weeks to 6 months. Two studies had a washout session after the intervention.

Ten studies (71.4%) [29,30,37,38,43,44,45,46,48,49] were randomized controlled trials (RCTs). Out of the 10 RCTs, 9 studies (64.3%) [29,30,37,38,43,45,46,48,49] included a placebo group in the study design, while the remaining study [43] was a cross-over study. Out of the 4 non-RCTs, a study [36] included a placebo group; a study [31] included a healthy control group; and a study [47] used other probiotics for the comparison group. The remaining study [42] did not have a comparison group.

**Table 1:** Studies concerning the use of probiotics in subjects with ASD.

Author (Year)	Country/Region	Type of Trial	Population	Intervention	Dose	CFU	Evaluation Tool	Findings	Limitations
Shaaban et al. (2017) [42]	Egypt	Prospective, open-label study	30 ASD 19 ♂ 11 ♀ Age 5–9 yrs	2 Lact strains (acidophilus, rhamnosus) + 1 Bifid strain (longum)	5 g/day for 3 months	5 × 10^8^	ATEC	Significant reductions in total ATEC scores, as well as improvements across all four subdomains: speech/language/communication, sociability, sensory/cognitive awareness, and health/physical/behavior.	Small sample size; no control group; unblinded study
Liu et al. (2019) [29]	Taiwan	Randomized, double-blinded, placebo-controlled trial	80 ASD (39 with PRO, 41 with PLA) ♂ ♀ missing Age 7–15 yrs	*Lactobacillus plantarum* PS128 with microcrystalline cellulose as carrier to be stored at 4–8 °C	1 capsule/day for 4 weeks	3 × 10^10^	ABC-T SRS SNAP-IV CGI	SNAP-IV-opposition/defiance and SNAP-IV-total score improved significantly in subjects aged 7–12 years in PS128 group. Only significant when stratified by age.	Short intervention period
Niu et al. (2019) [36]	China	Nonrandomized, unblinded, placebo-controlled trial	65 ASD (37 with PRO, 28 with PLA) ♂ ♀ missing Age 3–8 years	Probiotic strains not reported + ABA training	6 g/day for 4 weeks	3.6 × 10^9^	ATEC	ATEC total and all subdomains improved significantly in the probiotic group. Improvement in ASD groups without GI events was higher than those with GI events. (86.7% vs. 78.9%)	Small sample size; short intervention period; unblinded study
Arnold et al. (2019) [43]	United States	Randomized, double-blinded, controlled clinical trial	10 ASD (6 PRO then PLA, 4 PLA then PRO) 6 ♂ 4 ♀ Age 3–12 yrs	*Strep thermophilus* + 3 Bifid strains (breve, longum, infantis) + 4 Lact strains (acidophilus, plantarum, para-casei, delbrueckii subsp. bulgaricus)	1 packet/day for 8 weeks → 3 weeks washout → 8 weeks placebo Option for 2 packets/day at 4-week or 15-week visit if no effect was noted	9 × 10^11^ per packet	ABC SRS	Each outcome measure showed improvement over baseline, with probiotic phase showing more improvement than placebo phase, but not statistically significant.	Small sample size; no placebo-only control group; short intervention period
Santocchi et al. (2020) [44]	Italy	Randomized, double-blinded, placebo-controlled trial	85 ASD (42 with PRO, 43 with PLA) 71 ♂ 14 ♀ Age 18–72 mths	*Strep thermophilus* + 3 Bifid strains (breve, longum, infantis) + 4 Lact strains (acidophilus, plantarum, para-casei, delbrueckii subsp. bulgaricus) dissolved directly at mouth or in a cold, not carbonated liquid	2 packets/day in the first mth and 1 packet/day for the next 5 mths	4.5 × 10^11^ per packet	ADOS-CSS RRB VABS-II SCQ	In group without GI symptoms, ADOS-CSS scores (total and social affect) significantly decline in the probiotic group. In group with GI symptoms, VABS-II increased significantly in probiotic group in all subscales (receptive skills, domestic skills, and coping skills) in probiotic group. Only significant when segregated by GI symptoms.	High dropout rate; small age range
Mensi et al. (2021) [47]	Italy	Open-label, non-randomized clinical trial	131 ASD (105 with LP, 26 with OP) 112 ♂ 19 ♀ 86.1 ± 41.1 mnths (7.2 ± 3.4 yrs)	*Lactobacillus plantarum* PS128	1 packet/day (BW < 30 kg) or 2 packets/day (BW ≥ 30 kg) for 6 mths	3 × 10^10^ per packet	CGI	CGI-Improvement of 1–3 in 77.1% of all patients; correlation between younger age and improvement is observed (R = 0.283). CGI-I of 1–3 was reported for 91 patients of the LP group (86.7%).	No randomization; uneven number of participants in LP and OP groups; no placebo group; unblinded study
Sherman et al. (2022) [48]	Taiwan	Randomized, double-blinded, placebo-controlled trial	35 ASD (18 with PRO, 17 with PLA) 26 ♂ 9 ♀ 3–20 yrs	*Lactobacillus plantarum* PS128	6 × 10^10^ CFUs/day for 16 weeks	6 × 10^10^	SRS ABC-2 CGI	In probiotic group, changes in CGI-I, ABC-2 total score, and stereotypic behavior sub-score were positively correlated with baseline titers of anti-lysoganglioside GM1. Change in ABC-2 inappropriate speech sub-score was positively correlated with baseline titers of anti-dopamine receptor D1 in probiotic group. Change in SRS motivation sub-score was positively correlated with baseline titers of anti-tubulin in probiotic group. Change in serum GFAP concentration was positively correlated with baseline ABC-2 inappropriate speech sub-score in probiotic group. Change in Shannon index of gut microbiome was positively correlated with baseline CGI-S, SRS total score, SRS subscales (cognition and motivation), and ABC-2 subscales (hyperactivity/noncompliance and social withdrawal)	Small sample size; abundance of correlational tests; wide age range
Guidetti et al. (2022) [38]	Italy	Randomized double-blinded crossover clinical trial	61 ASD (30 with PRO then PLA, 31 with PLA then PRO) 50 ♂ 11 ♀ Age 2–16 yrs	3 Lact strains (fermentum LF10, salivarius LS03, plantarium LP01) + 5 Bifid longum strains (DLBL07–DLBL11)	2 sachets/day at first mth 1 sachet/day for 2 mths	1 × 10^10^ per sachet	PSI VABS PEP 3rd edition ASRS	Significant improvement in communication skills in VABS and receptive language. Significant improvement in PSI for both parents	Small sample size
Guiducci et al. (2022) [45]	Italy	Randomized double-blinded placebo-controlled trial	63 ASD (31 with PRO, 32 with PLA) ♂ ♀ missing Age 3–5 yrs	*Strep thermophilus* + 3 Bifid strains (breve, longum, infantis) + 4 Lact strains (acidophilus, plantarum, para-casei, delbrueckii subsp. bulgaricus)	2 packets/day in the first mth and 1 packet/day for the next 5 mths	4.5 × 10^11^ per packet	ADOS	Children in the “ADOS Total Score Improved” Group in the Probiotic group showed the highest 25(OH)D status at 29.9 ± 9.9 ng/mL. 25(OH)D below 30 ng/mL carries 5.6× higher risk of no improvement in ADOS.	No statistical analysis of ADOS score between treatment and control group; small sample size
Billeci et al. (2023) [46]	Italy	Randomized, double-blinded, placebo-controlled trial	46 ASD (26 with PRO, 20 with PLA) 35 ♂ 11 ♀ Age 18–72 mths	*Strep thermophilus* + 3 Bifid strains (breve, longum, infantis) + 4 Lact strains (acidophilus, plantarum, para-casei, delbrueckii subsp. bulgaricus)	2 packets/day in the first mth and 1 packet/day for the next 5 mths	4.5 × 10^11^ per packet	ADOS-2 CARS SCQ RBS-R CBCL VABS-II	No significant changes in all the scales measured.	Small sample size; focused on EEG findings
Mazzone et al. (2023) [37]	USA	Randomized, double-blinded, placebo-controlled trial	43 ASD (21 with PRO, 22 with PLA) 35 ♂ 8 ♀ Age 6.23 ± 1.15 years	*Limosilactobacillus reuteri* DSM17938 and ATCC PTA 6475	2 tablets/day for 6 mths	2 × 10^8^ per tablet	ADOS-2 SRS CBCL ABAS-2	The total T score and social communication domain score of SRS significantly improved. Social adaptive composite score in ABAS-2 significantly improved.	Small sample size
Li et al. (2024) [31]	China	Open-label, single-arm pilot study	53 ASD (49 ♂ 4 ♀); age 6.49 ± 3.08 yrs 45 TD (41 ♂ 4 ♀) Age 7.68 ± 3.61 yrs	*Bifid animalis* subsp. lactis Probio-M8 + Balanced diet (30–40 kcal/kg with 40% carbohydrate, 30% fats, 30% proteins)	Dry powder 2 g/day for 12 weeks	1 × 10^11^	CARS	CARS scores significantly decreased by 15.22%.	No placebo control group; unblinded study; high dropout rate (19 out of 72)
Rojo-Marticella et al. (2025) [49]	Spain	Randomized, double-blinded, placebo-controlled trial	42 ASD (21 with PRO, 21 with PLA) 35 ♂ 7 ♀ Age 5–16 yrs	*Lactiplantibacillus plantarum* + *Levilactobacillus brevis* (1:1 ratio)	1 sachet/day for 12 weeks	1 × 10^9^	CPT-3/ K-CPT 2 SRS-2	Hyperactivity–impulsivity symptoms significantly improved (7.20 ± 3.76) in younger children (5–9 years old).	Small sample size
Khanna et al. (2025) [30]	India	Randomized single-blinded placebo-controlled trial	180 ASD (90 with PRO, 90 with PLA) 139 ♂ 41 ♀ 2–9 years	*Saccharomyces boulardii* + 4 Bifid strains (breve, brevebifidum, animalis lactis, longum infantis) + 6 Lact strains (reuteri, rhamanosus, planetarium, acidophilus, casie, delbrueckii bulgaricus) + *Streptococcus thermophilus* mixed with 50 mL of lukewarm milk or water	5 g/day for 3 mths	9 × 10^9^	SRS-2 ABC-2	Significant improvements in SRS-2 total T score and significant reduction in percentage of children with severe SCI and RRBI subscores. Significant reductions of percentage of children with severe social withdrawal, stereotypic behavior, hyperactivity, and inappropriate speech.	Not double-blinded

Abbreviations: ABAS-2: Adaptive Behavior Assessment System—Second Edition; ABC: Autism Behavior Checklist; ABC-2: Aberrant Behavior Checklist—Second Edition; ABC-T: Autism Behavior Checklist—Taiwan Version; ADOS: Autism Diagnostic Observation Schedule; ADOS-2: Autism Diagnostic Observation Schedule, Second Edition; ADOS-CSS: Autism Diagnostic Observation Schedule—Calibrated Severity Score; ASD: Autism Spectrum Disorder; ASRS: Autism Spectrum Rating Scales; ATEC: Autism Treatment Evaluation Checklist; CARS: Childhood Autism Rating Scale; CBCL: Child Behavior Checklist; CFU: colony-forming units; CGI: Clinical Global Impressions Scale; CPT-3: Conners Continuous Performance Test—Third Edition; EEG: Electroencophalogram; K-CPT 2: Kiddie Conners Continuous Performance Test—Second Edition; LP: *Lactobacillus plantarum*; OP: other probiotic; PEP: psychoeducational profile; PLA: placebo; PRO: probiotic; RBS-R: Repetitive Behavior Scale—Revised; RRB: restricted and repetitive behaviors; SCQ: Social Communication Questionnaire; SNAP-IV: Swanson, Nolan and Pelham Rating Scale—Version IV; SRS: Social Responsiveness Scale; SRS-2: Social Responsiveness Scale—Second Edition; VABS: Vineland Adaptive Behavior Scales; VABS-II: Vineland Adaptive Behavior Scales—Second Edition; ♂: male; ♀: female.

### 3.2. Study Quality

**Figure 2:**
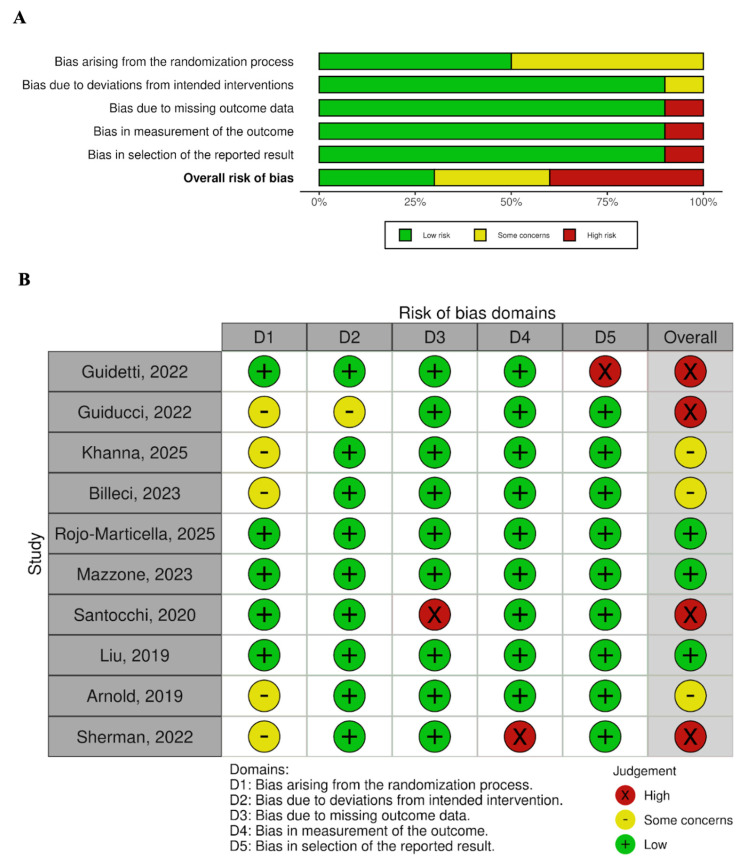
Risk of bias graph (**A**) and risk of bias summary (**B**) for RCTs [29,30,37,38,43,44,45,46,48,49].

A risk of bias assessment was done for 10 RCTs and 4 non-randomized studies. Figure 2 and Table 2 show the results of methodological quality assessment of randomized controlled trials and non-randomized studies.

RCTs have a lower risk of bias compared to nonrandomized studies. Three RCT studies have a low risk of bias, three RCT studies have some concerns for risk of bias, and four RCT studies have a high risk of bias. All nonrandomized studies have at least a serious risk of bias, with one of them having a critical risk of bias. Among the domains of RoB 2, the domain that raises the most concerns is the randomization process, as the papers do not report the process and method of randomization. In nonrandomized studies, the most problematic methodological areas are due to confounding factors and measurement of the outcome. Most nonrandomized studies did not make enough effort to control for confounding factors, such as baseline GI symptoms and dietary variation. Additionally, all of the nonrandomized studies were not blinded, making the clinician- or parent-tasked evaluation of autism patients treated with probiotics prone to risk of bias.

### 3.3. Main Findings

The majority of the studies reported improvement in the core symptoms of ASD. However, only eight studies’ findings were significant in improvement in different domains of core ASD symptoms. One of the studies reported significant findings only when the probiotic group was separated into a GI group and a non-GI group. Another study only had significant results when participants were stratified based on age group. One study reported significant improvement in core symptoms correlated with a change in specific metabolites after participants were treated with probiotics. Five out of 15 studies (33.3%) reported direct improvement of ASD core symptoms after probiotic intervention without subgroup division or any stratification.

Among the five domains in the core symptoms of ASD analyzed, the domains which were most reported to have significant improvement were “Social & Communication” (n = 6); other domains included “Restrictive & Repetitive Behavior” (n = 3), “Adaptive Functioning” (n = 3) and “Sensory & Cognitive Processing” (n = 2). *Lactobacillus* spp. was the most used bacterial strain in studies with significant improvement in core ASD symptoms (n = 6), followed by *Bifidobacterium* spp. (n = 5), *Streptococcus* spp. (n = 2), *Lactobacillus plantarum* PS128 (n = 2), *Lactobacillus reuteri* (n = 2), and *Saccharomyces boulardii* (n = 1).

The intervention duration most commonly used was “3-month” (n = 6) with four studies reporting significant improvement (66.7%), followed by “6-month” (n = 5) with two studies had significant improvement (40%), “4-week” (n = 2) with one reporting significant improvement (50%), “16-week” (n = 1) with significant improvement (100%), and “8-week” (n = 1) without significant improvement.

There were two studies that reported significant improvement in all domains of the core ASD symptoms. Niu et al. [36] tested unspecified probiotics combined with Applied Behavior Analysis (ABA) for 4 weeks and found significant improvements in total ATEC scores and all subdomains, including “Communication”, “Social”, “Sensory/Cognitive Functioning”, and “Health and Behavior”, with greater improvement in children without GI symptoms. Shaaban et al. [42] reported a similar finding across all subdomains of ATEC scores using *Lact. acidophilus*, *Lact. Rhamnosus*, and *Bifid. longum* at 0.5 billion CFUs/day for 3 months.

### 3.4. Main Findings According to Domains of Core ASD Symptoms

The main findings according to the domains of core ASD symptoms are reported in Table 3. Apart from the two studies mentioned above that observed improvement in all domains, another four studies found significant improvement in the “Social & Communication” domain. Guidetti et al. [38] found significant improvement in the receptive language scale and the communication domain of VABS. Khanna et al. [30] observed a significant reduction of SRS-2 total T score in the probiotic group, as well as a significant reduction in symptom severity in the social communication and interaction (SCI) domain. Meanwhile, Mazzone et al. [37] found a significant reduction in SRS total T score and social communication subscale after *L. reuteri* treatment. Moreover, the social adaptive composite score in ABAS-2, which can be considered under the “Social” domain of core ASD symptoms, was also significantly improved in the probiotic group. Santocchi et al. [44] reported that the total ADOS-CSS score and the social affect domain significantly declined in subjects without GI symptoms in the probiotic group. They also reported that those who received probiotics with baseline GI symptoms showed a significant increase in the receptive skills domain of VABS-II. The subdomains across different scales that were significantly improved are:

- Social communication in SRS [37] and SRS-2 [30];
- Social adaptive skills in ABAS-2 [37];
- Social affect in ADOS-CSS [44];
- Receptive skills in VABS [38] and VABS-II [44];
- Speech/language/communication and sociability in ATEC [36,42];
- Communication in VABS [38];
- Social withdrawal or lethargy and inappropriate speech in ABC-2 [30].

For the “Restrictive & Repetitive Behavior” domain, apart from the studies conducted by Niu et al. [36] and Shaaban et al. [42], which reported improvement in all domains, Khanna et al. [30] also observed significant improvement in symptom severity in the restricted, repetitive behaviors and interests (RRBI) domain of SRS-2 after a 3-month probiotic intervention. The subdomains across different scales that were significantly improved are:

- Stereotypic behavior in ABC-2 [30];
- Health/physical/behavior in ATEC [36,42].

For the “Adaptive Functioning/Daily Living Skills” domain, Santocchi et al. [44] reported a significant increase in the domestic skills and coping skills domain of VABS-II in subjects who received probiotics with baseline GI symptoms. The subdomains across different scales that were significantly improved are:

- Domestic skills and coping skills in VABS-II [44];
- Health/physical/behavior in ATEC [36,42].

For the “Sensory & Cognitive Processing” domain, there were no other studies that reported a significant change in this domain. The subdomains across different scales that were significantly improved are:

- sensory/cognitive awareness in ATEC [36,42].

**Table 3:** Main findings of the studies according to the domains of core ASD symptoms.

Domain	Risk of Bias	Author	Intervention	Main Findings	*p* Value
SC	Low	Mazzone et al. [37]	*L. reuteri* DSM17938 and ATCC PTA 6475 2 tablets/day (0.4 billion CFUs in total) for 6 mths	Social communication subscore of SRS improved from 12.3 ± 5.1 to 10.3 ± 3.5 in the probiotic group. Social adaptive composite score of ABAS-2 improved from 65.60 ± 12.73 to 69.70 ± 14.45 in the probiotic group.	*p* = 0.005 *p* = 0.018
	Some concerns	Khanna et al. [30]	*Saccharomyces boulardii* + 4 Bifid strains (breve, brevebifidum, animalis lactis, longum infantis) + 6 Lact strains (reuteri, rhamanosus, planetarium, acidophilus, casie, delbrueckii bulgaricus) + *Streptococcus thermophilus* 5 g/day (9 billion CFUs in total) for 3 mths	SRS-2 total T score reduced from 76.59 ± 6.76 to 70.49 ± 8.51. Percentage of children with severe SCI T score of SRS decreased from 58.90% to 16.70% of total participants in the probiotic group. Percentage of children with severe social withdrawal or lethargy and inappropriate speech of ABC-2 decreased for 40% and 32.22%, respectively.	*p* = 0.340 *p* < 0.001 *p* < 0.001
	High	Guidetti et al. [38]	3 Lact strains (fermentum LF10, salivarius LS03, plantarium LP01) + 5 Bifid longum strains (DLBL07–DLBL11) 2 sachet/day (20 billion CFUs in total) at 1st mth, 1 sachet/day (10 billion CFUs in total) onwards for total of 3 mths	Significant improvement in communication skills in VABS (+11.3 ± 4.6) and receptive language (+18 ± 8.44)	*p* = 0.01 *p* = 0.03
		Santocchi et al. [44]	*Strep thermophilus* + 3 Bifid strains (breve, longum, infantis) + 4 Lact strains (acidophilus, plantarum, para-casei, delbrueckii subsp. bulgaricus) 2 packets/day (900 billion bacteria) in the first mth and 1 packet/day (450 billion bacteria) for the next 5 mths	Social-affect subscale in ADOS-CSS improved from 6.09 ± 2.00 to 4.95 ± 1.56 in the group of participants without baseline gastrointestinal symptoms. Receptive scale in VABS-II improved from 4.78 ± 3.03 to 7.11 ± 3.14 in the group of participants with baseline gastrointestinal symptoms.	*p* = 0.027 *p* = 0.010
	Serious	Niu et al. [36]	Probiotic strains not reported + ABA training 6 g/day (36 billion CFU in total) for 4 weeks	Speech/language/communication score of ATEC improved from 14.6 to 13.2 (standard deviation not reported). Sociability score of ATEC improved from 16.6 to 14.3 (standard deviation not reported)	*p* < 0.001 *p* < 0.001
		Shaaban et al. [42]	2 Lact strains (acidophilus, rhamnosus) + 1 Bifid strain (longum) 5 g/day (0.5 billion CFUs in total) for 3 months	Speech/language/communication score of ATEC improved from 18.60 ± 4.61 to 18.30 ± 4.59 Sociability score of ATEC improved from 17.50 ± 4.75 to 15.73 ± 4.28	*p* = 0.017 *p* = 0.001
RRB	Some concerns	Khanna et al. [30]	*Saccharomyces boulardii* + 4 Bifid strains (breve, brevebifidum, animalis lactis, longum infantis) + 6 Lact strains (reuteri, rhamanosus, planetarium, acidophilus, casie, delbrueckii bulgaricus) + *Streptococcus thermophilus* 5 g/day (9 billion CFUs in total) for 3 mths	Percentage of children with severe RRBI T score of SRS decreased from 57.80% to 15.60% of total participants in the probiotic group. Percentage of children with severe stereotypic behavior subscale of ABC-2 decreased for 37.77%.	*p* < 0.001 *p* < 0.001
	Serious	Niu et al. [36]	Probiotic strains not reported + ABA training 6 g/day (36 billion CFU in total) for 4 weeks	Health/physical/behavior score of ATEC improved from 17.8 to 14.2. (standard deviation not reported).	*p* < 0.001
		Shaaban et al. [42]	2 Lact strains (acidophilus, rhamnosus) + 1 Bifid strain (longum) 5 g/day (0.5 billion CFUs in total) for 3 months	Health/physical/behavior score of ATEC improved from 36.83 ± 8.32 to 27.10 ± 5.83.	*p* < 0.001
AF/DLS	High	Santocchi et al. [44]	*Strep thermophilus* + 3 Bifid strains (breve, longum, infantis) + 4 Lact strains (acidophilus, plantarum, para-casei, delbrueckii subsp. bulgaricus) 2 packets/day (900 billion bacteria) in the first mth and 1 packet/day (450 billion bacteria) for the next 5 mths	Domestic skills in VABS-II improved from 9.44 ± 5.50 to 12.66 ± 2.74 in the group of participants with baseline gastrointestinal symptoms. Coping skills in VABS-II improved from 9.11 ± 4.01 to 10.22 ± 2.17 in the group of participants with baseline gastrointestinal symptoms.	*p* = 0.047 *p* = 0.012
	Serious	Niu et al. [36]	Probiotic strains not reported + ABA training 6 g/day (36 billion CFU in total) for 4 weeks	Health/physical/behavior score of ATEC improved from 17.8 to 14.2 (standard deviation not reported).	*p* < 0.001
		Shaaban et al. [42]	2 Lact strains (acidophilus, rhamnosus) + 1 Bifid strain (longum) 5 g/day (0.5 billion CFUs in total) for 3 months	Health/physical/behavior score of ATEC improved from 36.83 ± 8.32 to 27.10 ± 5.83.	*p* < 0.001
SCP	Serious	Niu et al. [36]	Probiotic strains not reported + ABA training 6 g/day (36 billion CFU in total) for 4 weeks	Sensory/cognitive awareness score of ATEC improved from 18.1 to 16.4 (standard deviation not reported).	*p* = 0.003
		Shaaban et al. [42]	2 Lact strains (acidophilus, rhamnosus) + 1 Bifid strain (longum) 5 g/day (0.5 billion CFUs in total) for 3 months	Sensory/cognitive awareness score of ATEC improved from 20.47 ± 4.26 to 19.67 ± 4.17.	*p* = 0.026

Abbreviations: ABAS-2: Adaptive Behavior Assessment System—Second Edition; ABC-2: Aberrant Behavior Checklist—Second Edition; ADOS-CSS: Autism Diagnostic Observation Schedule—Calibrated Severity Score; AF/DLS: adaptive functioning/daily living skills; ATEC: Autism Treatment Evaluation Checklist; CFU: colony-forming units; RRB: restrictive and repetitive behaviors; RRBI: Restricted and Repetitive Behaviors Index; SC: social and communication; SCP: sensory and cognitive processing; SRS: Social Responsiveness Scale; VABS-II: Vineland Adaptive Behavior Scales—Second Edition.

### 3.5. Findings from Other Studies Not According to the Domain

There is an open-label study that reports a significant reduction in total CARS score. However, the results of domain scores were not reported [31]. Another open-label, nonrandomized study tested *Lact. plantarum* PS128 used CGI to assess the severity and improvement of ASD in subjects and found a significant reduction in CGI-severity in the probiotic group [47]. Although Liu et al. did not find improvement in autism-related questionnaires (ABC-T and SRS), they noticed that SNAP-IV opposition/defiance and SNAP-IV total score improved significantly in younger subjects (7–12 years old) when the subjects were stratified by age [29].

Interestingly, some studies evaluated the association of core ASD symptoms with the baseline or changes in specific body metabolites after probiotic intervention, demonstrating an indirect link between the effects of probiotic intervention and core ASD symptoms. Guiducci et al. [45] reported significant positive correlations between the baseline serum 25-hydroxyvitamin D level and reductions in ADOS scores in the probiotic group. Additionally, the same study that reported children treated with probiotics who had an improved ADOS total score showed the highest baseline serum 25-hydroxyvitamin D level. Sherman et al. [48] reported that changes in ABC-2 and CGI-I scores were positively correlated with baseline titers of anti-lysoganglioside GM1 in subjects treated with probiotics. Apart from that, they also found that baseline titers of anti-dopamine receptor D1 and anti-tubulin were positively correlated with the change in sub-scales of ABC-2 and SRS.

Finally, the remaining three studies did not report significant improvement in the core ASD symptoms after probiotic intervention [43,46,49].

### 3.6. Risk of Bias and Reported Improvements

The summary of the study’s risk of bias against the reported main findings was recorded in Table 4. Among randomized controlled trials (RCTs) with low risk of bias, only Mazzone et al. [37] reported significant improvement in the social communication (SC) domain, while no significant improvements were observed in other domains. RCTs with some concerns demonstrated improvement in the SC and RRB domains by Khanna et al. [30], whereas those with high risk of bias showed improvements in SC and AF/DLS domains, with Santocchi et al. [44] contributing to both. Non-RCTs with serious risk of bias reported improvements across multiple domains, including SC, RRB, AF/DLS, SCP, and overall scores, particularly in studies by Niu et al. [36] and Shaaban et al. [42]. However, studies with a critical risk of bias demonstrated improvement only in overall scores, as reported by Mensi et al. [47]. Notably, several studies across all risk categories reported no significant improvement.

### 3.7. Evaluation Tools of ASD Across Studies

The summary of ASD evaluation tools used and the results reported are recorded in Table 5. The studies included in this review utilized a variety of evaluation tools to assess the effectiveness of interventions in pediatric patients with Autism Spectrum Disorder (ASD). The most-used tools among the studies were the SRS/SRS-2 (n = 6), ATEC (n = 4), and ADOS/ADOS-2 (n = 4). Objective clinician-administered assessments, including ADOS/ADOS-2 and CARS, generally reported lower rates of significant improvement, with only 50% of measures demonstrating improvement, often limited to specific subgroups or indirect effects. Mixed evaluations combining clinician assessment and caregiver reporting, such as VABS/VABS-II, showed higher responsiveness, with significant improvement reported in 75% of cases, particularly in targeted intervention groups. Subjective caregiver-reported tools, including ABAS-2, ATEC, ABC/ABC-2, and SRS/SRS-2, demonstrated moderate overall improvement, with 58.33% of outcomes showing significant change.

### 3.8. Attempts to Control Confounding Factors

Across the included trials evaluating probiotic interventions for core symptoms of autism spectrum disorder (ASD), the authors employed multiple methodological strategies to mitigate potential confounding. The summary of the authors’ attempts to control the confounding factors is recorded in Table 6. Dietary factors were commonly addressed, with several studies excluding participants on special diets [30,37,42,44,45,46], implementing food diaries or diet logs [37,43,46], or formally assessing diet quality before and/or after intervention [30,36,42,44,49]. Meanwhile, Li et al. [31] incorporated a uniform balanced carbohydrate diet together with probiotic supplementation.

Nutritional status was variably controlled through baseline assessments [29,31,42,44,45,46,49] and the exclusion of malnourished children [31,43]. Perinatal factors were addressed in several studies by excluding children with severe prematurity, birth asphyxia, or perinatal injury [30,44,45,46], while breastfeeding history was documented in a minority of trials [30,38,44]. Concomitant medication use was the most consistently controlled confounder. Most studies excluded recent or ongoing use of antibiotics, probiotics, psychotropic agents, anti-inflammatory drugs, or other gastrointestinal therapies within predefined washout periods, and some prospectively monitored medication use throughout the study period. A few trials also recorded ongoing behavioral or speech therapies and required stability of such interventions during follow-up [30,37]. Nevertheless, the extent and rigor of confounder control varied considerably across studies, with some providing limited or no explicit strategies, thereby introducing potential residual confounding.

**Table 6:** Summary of attempts of authors in controlling confounding factors.

Author (Year)	Diet Status	Nutritional Status	Birth History	Breastfeeding	Other Medication Use/Therapies
Shaaban et al. (2017) [42]	Assessed at baseline; excluded patients on special diet.	Assessed at baseline and after intervention	-	-	Excluded patients on anti-fungal, antibiotics, psychiatric medications within the preceding 3 months. No other therapies 2 weeks before and during the study period.
Liu et al. (2019) [29]	-	Assessed at baseline	-	-	Excluded patients on antibiotics, yogurt, or probiotic products in preceding 2 weeks. Excluded patients on antibiotics during the study period. Asked to refrain from yogurt or probiotic products during the study period.
Niu et al. (2019) [36]	Assessed before and after intervention.	-	-	-	Excluded patients on antibiotic, probiotics, or other GI treatments in preceding 1 month.
Arnold et al. (2019) [43]	Diet logs were completed for 3 days before each stool sample collection.	Excluded patients with weight or height less than 3rd percentile for age	-	-	Excluded patients on antibiotics or chronic anti-inflammatory use within the preceding 2 months.Excluded patients on probiotics within the preceding 6 months.
Santocchi et al. (2020) [44]	Assessed at baseline. Excluded patients on special diets.	Assessed at baseline	Excluded severe premature birth, birth asphyxia, or perinatal injuries.	Assessed	Administration of antibiotics, NSAIDs, paracetamol, steroids, psychotropic drugs, and other drugs was recorded during the study period.
Mensi et al. (2021) [47]	-	-	-	-	-
Sherman et al. (2022) [48]	-	-	-	-	Excluded patients on psychotropic medications.Excluded patients who had received oxytocin or probiotic treatment within the last 4 weeks.Excluded patients on antibiotics during study period.
Guidetti et al. (2022) [38]	Parents were informed to try limiting excessive food consumption or any food abuse.	-	Assessed	Assessed	Excluded patients on long-term antibiotics, other probiotics, cortisone, anti-inflammatory drugs, amiodarone, valproate, and statins one month before and during the enrollment.Use of antibiotics less than 10 days during the enrollment was reported.
Guiducci et al. (2022) [45]	Excluded patients on special diets	Assessed at baseline	Excluded birth asphyxia, severe premature birth, or perinatal injuries.	-	-
Billeci et al. (2023) [46]	Weekly food diaries filled in by caregivers. Excluded patients on special diets.	Assessed at baseline and after intervention	Excluded birth asphyxia, severe premature birth, or perinatal injuries.	-	Detailed treatment data was assessed at baseline; concomitant drug consumption monitored by caregiver’s interview.
Mazzone et al. (2023) [37]	Weekly diary to report modification in daily diet; excluded patients on special diets.	-	-	-	Weekly diary reporting concomitant medications (antibiotics, anti-inflammatory); allowed to continue the concomitant therapies, such as behavioral and speech therapy, as long as no modification of intervention during the study period.
Li et al. (2024) [31]	Medium-carbohydrate diet incorporated in intervention.	Excluded patients diagnosed malnutrition.Assessed at baseline.	-	-	Excluded patients on immunosuppressants, antibiotics, probiotics, prebiotics, or postbiotics within 1 month before or during the intervention.
Rojo-Marticella et al. (2025) [49]	Diet quality assessed before and after the intervention.	Assessed before and after intervention.	-	-	Excluded patients on antibiotic at the start of intervention.
Khanna et al. (2025) [30]	Excluded patients on special diets; Assessed at baseline (semisolid or solid).	Assessed at baseline.	Excluded birth asphyxia, severe premature birth, or perinatal injuries.	Assessed at baseline (breastfed, formula-fed, or both)	Patients with ongoing behavioral therapy recorded at baseline.Excluded patients on antibiotics within 2 months or long-term use.Excluded patients on psychotropic medications within 3 months.Excluded patients on probiotic supplement within 6 months.

## 4. Discussion

### 4.1. Evidence of Probiotics on Improving Core ASD Symptoms

From the studies examined, probiotic supplementation showed significant improvement of core ASD symptoms. A similar observation was reported by other systematic reviews [50,51,52]. Most included studies were assessed as having a high, serious, or critical risk of bias, primarily due to inadequate control of confounding factors, lack of detail of the randomization method, and selective reporting, which significantly compromises internal validity. Consequently, despite some positive findings, the overall evidence supporting probiotics in improving core ASD symptoms remains weak and unreliable. However, other systematic reviews show half of the studies have a lower risk of bias, showing a discrepancy in reporting risk of bias despite similar papers being retrieved. This could be due to subjective interpretation of methodological reporting, access to supplementary information, and reviewer judgement [50].

Probiotics were found to alleviate autism by the restoration of neurotransmitter balance. It was proposed that probiotics promote improvement in excitatory/inhibitory imbalance, which is typically found in the brain activity of ASD patients, by modulating neuroactive compounds [53,54]. Strains such as *L. reuteri* and *L. helveticus* increase the expression of GABA and its receptors while decreasing levels of glutamate in the brain [53,55,56]. Meanwhile, probiotics also regulate the metabolism of serotonin (5-HT) and increase oxytocin levels in the brain [53,57]. Meanwhile, probiotics may reduce gut inflammation and improve immune functions. Probiotic treatment has been shown to decrease pro-inflammatory cytokines such as IL-1, IL-6, and TNF-α [58,59]. Probiotics also restore gut-barrier integrity, thus preventing harmful substances and pro-inflammatory markers from entering the bloodstream [60]. Lastly, probiotics’ action in modulating the gut microbiome profile indirectly regulates serum and brain metabolites, such as short-chain fatty acids (SCFAs), 5-aminovaleric acid, and choline [61,62]. In short, probiotics may improve autism by restoring neurotransmitter balance, reducing gut inflammation, restoring gut-barrier integrity, and regulating metabolites.

### 4.2. Effect of Probiotics on “Social and Communication” Domain

The “Social and Communication” domain was reported to have the most significant improvement after probiotic intervention. However, it is important to note that most studies have a high risk of bias. In comparison, the other two studies [29,49] with a low risk of bias do not report significant improvement in this domain, making the strength of evidence relatively weak. On top of that, *L. reuteri* was the only consistent strain found to improve social and communication function. This limits the generalizability of probiotics from other strains to have a similar effect on children with ASD. In terms of evaluation of the tools used, most studies, including studies with lower risk [30,37], reported improvement in subjective reporting tools by caretakers. Subjective reporting tools were prone to expectation bias; two studies [36,42] were open-label studies, meaning the participants were not blinded.

Early evidence suggests that gut microbiota play an important role in the evolution of social architecture in animals. It is proposed that gut microbiota may influence neurodevelopment and social behavior programming across different species of animals [63]. A study in 2014 supported this hypothesis, as mice under germ-free rearing conditions displayed altered social preference and social avoidance compared to conventionally colonized mice. Additionally, a reversal of observed social deficits was seen when a new set of germ-free mice was colonized with fecal bacteria from a neurotypical counterpart [64]. The improvement in the social and communication domain is consistent with other preclinical studies [65,66,67], further strengthening the link found between gut microbiota and socio-communication deficits in ASD patients. In human studies, gut dysbiosis in ASD patients is found to be related to core social and communication deficits [68,69]. A meta-analysis of microbiota-based trials on ASD patients found that fecal microbiota transplantation (FMT) gives significant improvement in the social functioning of ASD patients [70], supporting the effect of microbiota modulation on social and communication function in humans.

The model of social deficit is a complex topic in the pathophysiology of ASD. Deficits in social motivation in ASD patients consist of disruption in social orienting, social seeking, liking, and social maintaining [71]. These deficits appear to be in biological disruptions of the orbitofrontal–striatal–amygdala circuitry and in the dysregulation of certain neuropeptides and neurotransmitters [71]. Modulation of gut microbiota in animal models has been proven to improve social deficit in ASD [65,66,67] by restoring oxytocin levels and increasing plasticity of the ventral tegmental area, a possible mechanism of how probiotics can alleviate social and communication deficits.

### 4.3. Effect of Probiotics on Other Domains

Aside from the “social and communication” domain, findings showing improvement in the other domains were present but scarce. Most studies reporting improvement in these domains have a higher risk of bias; improvements were reported in subjective reporting scales such as ATEC and SRS, making the findings at risk of expectation bias.

Most current evidence of the link between the gut–brain axis and stereotypic, repetitive behavior is found in preclinical studies. This is proven in animal study models as animals with stereotypic, repetitive behavior were found to have a reduced count of Bacteriodales in their gut microbiome [72], further proving the link between the gut–brain axis and stereotypical behaviors in ASD individuals. This marks the possibility that alteration of the gut microbiome could benefit core ASD symptoms, not only in the socio-communication domain. Trials of probiotics in ASD mouse models reveal improvement in repetitive behavior [60,73]. For instance, mice treated with B. fragilis had reduced stereotyped marble-burying behavior [60], and L-tyrosine-regulated microbiota reduced repetitive behaviors, aside from improving the social function of mice [74]. In a clinical study, specific strains in the gut microbiota are related to repetitive behavior of children with ASD [74], specifically *L. plantarum* and *B. longum*.

Lack of high-quality evidence in domains other than the social domain may be attributable to the heterogeneous etiology of ASD. As demonstrated by the preclinical studies above, specific strains of gut microbiome and probiotics are responsible for repetitive behavior in ASD mice. The complex interplay between the gut microbiome–brain axis and ASD symptomatology may contribute to differences in response between different domains of core ASD symptoms. It may be related to differences in neural circuits implicated in social and non-social behaviors. For instance, repetitive behavior in ASD is mediated by the cortico-basal ganglia–thalamic pathway [75,76]. Meanwhile, social deficit is due to abnormalities in the orbitofrontal–striatal–amygdala pathway and ventral tegmental area [71,77,78].

### 4.4. L. reuteri Related to Improvement in “Social & Communication” Domain

*L. reuteri* was examined by two RCTs [30,37]. The results showed significant improvement in SRS-2 and SRS. Interestingly, both studies have a lower risk of bias, having stronger evidence, although the mode of evaluation is by subjective reporting from a caretaker. An even more interesting finding is that, among all 14 studies, only these two studies used *L. reuteri*, giving it a current 100% chance to improve social and communication function in both RCTs [30,37]. Mazzone et al. [37] used *L. reuteri* as the only probiotic strain, while Khanna et al. mixed *L. reuteri* with other strains of probiotics. This finding highlights the role of *L. reuteri*, possibly producing better efficacy compared to other probiotic strains. Multiple preclinical studies [65,66,67] have found that *L. reuteri* played an important role in improving social and communication function in ASD rats. Across multiple rodent models, *L. reuteri* consistently shows neuroprotective, anti-inflammatory, and cognition-supporting effects, aside from improving social function in ASD [79,80,81]. *L. reuteri* engages the gut–brain axis by reshaping the microbiota, generating neuroactive metabolites (GABA, 5-HT, indoles, SCFAs), reinforcing barrier and immune homeostasis, and activating vagal–oxytocin and stress circuits [82,83,84].

From these two studies, Mazzone et al. [37] treated the participants for 6 months, and Khanna et al. [30] treated them for 3 months. This could possibly mean significant improvement may only be seen after at least 3–6 months of probiotic intervention. In terms of dosage, Mazzone et al. [37] used 0.4 billion CFUs daily and Khanna et al. [30] prescribed 9 billion CFUs daily. Based on these two high-quality studies, *L. reuteri* at a dose of 0.4 billion CFU daily with or without other strains of probiotics, administered for at least 3–6 months, shows evidence of potential benefit in social and communication domains in children with ASD, although further well-designed trials are required.

### 4.5. Role of Baseline Gastrointestinal Symptoms on Efficacy of Probiotics

Most of the studies (73.3%) measured the GI symptoms of the subjects. Studies [36,44] compared the improvement of core ASD symptoms between subjects with and without baseline GI symptoms. However, the results were variable and did not have a clear pattern. RCT conducted by social affect improvement was reported in subjects without GI symptoms and adaptive functioning improvement in subjects with GI symptoms [44]. Another study [36] found that the subjects without GI symptoms had more improvement in total ATEC score than those with GI symptoms. The variability of response could be explained by the neurobiological heterogeneity of ASD and possibly a more complex underlying gut–brain interaction mechanism that had yet to be understood. Recent pharmacological trials have proven that a single medication would not give the same benefit to every ASD child [85,86]. Another hypothesis is the correlation between the presence of GI symptoms and the severity of ASD, indicating the underlying reason behind why those without GI symptoms respond better to probiotic treatment [32]. Future studies should investigate more on the differences in response between ASD children with and without GI symptoms towards probiotic intervention.

### 4.6. Choice of ASD Evaluation Tools on Reported Efficacy of Probiotics

The most consistent improvements were reported using subjective caregiver-reported measures such as ATEC, ABC/ABC-2, and SRS/SRS-2. These instruments are the most popular choice among the studies, probably due to the lower difficulty and expertise needed to conclude the score. Evidence of significant improvement in studies with low or some concerns of risk of bias is seen more among these instruments compared to objectively evaluated tools. The underlying reason could be due to expectation bias from the caregivers. Subjective reporting measures are quicker and more convenient ways to assess treatment outcomes. However, they come with notable limitations that can distort the internal validity of studies. Caregivers may exaggerate or minimize symptoms, as they might inaccurately remember past behavior or answer in ways that are believed to be socially acceptable [87,88].

The most popular evaluation tools among the studies examined were SRS, ATEC, and ADOS. SRS is mainly used to screen and measure social responsiveness. Although having good sensitivity and specificity for ASD, SRS is not a diagnostic tool and has a high risk of being influenced by the rater [89,90]. ATEC is mainly indicated in the monitoring of treatment effects in ASD children. Although ATEC is prone to subjective reporting bias, it is a free and quick assessment tool and correlates with CARS, which is an ASD diagnostic tool, making it easily accessible and performable while having good internal consistency [22,91]. Finally, ADOS is used with structured observation by trained clinicians for ASD diagnosis, which is considered the “gold standard”, and ADOS is widely validated. ADOS, having high sensitivity and specificity, should be used in an ideal setting, but it is costly and time-consuming while requiring extensive training, rendering it impractical in settings with limited time and resources [19,92,93]. In future studies, ADOS is recommended when time and resources permit it. ATEC and SRS are popular alternatives to ADOS, but subjective reporting bias should be addressed and controlled as much as possible.

Though objectively evaluated tools like ADOS are considered the gold standard, perceived behavioral change may not reflect modification of core neurodevelopmental features. Behavioral improvement can occur while core neural differences persist as compensation and adaptation of behavior through social-skills training [88,94]. To reflect core neurodevelopmental features, imaging or biomarker analysis would be needed to tie changes in behavior to modification of neuroanatomical and biochemical development. A longitudinal MRI could be used to detect differences in cortical thickness, surface area, and volume in social and communication regions, which are linked to changes in adaptive behavior in autism [95].

### 4.7. Confounding Factors

Dietary habits, nutrition, medications (especially antibiotics), very preterm infants, and breastfeeding were known for altering gut microbiota [96,97,98,99,100]. Controlling these confounding factors was crucial to ensure internal validity and establish true cause-and-effect relationships of probiotic supplementation on core ASD symptoms. Diet status, nutritional status, and medication use were controlled in most of the studies. However, birth history and breastfeeding were only controlled in a minority of studies. Although diet status is controlled by excluding those on special diets, the studies did not incorporate a uniform diet for all participants to eliminate variable bias attributed to diet. Ideally, a uniform diet should be administered to both the intervention and control groups. The diet is recommended to include high-fiber, antioxidants, omega-3 fatty acids, and phytochemicals, which reduce gut inflammation [101,102]. Supplementation may also be considered, such as folic acid, vitamin B6, vitamin D, and L-Carnitine, which are proven to be beneficial for ASD patients that are linked to an anti-inflammatory profile [103].

Nutritional status was controlled in most of the studies, and all studies had no significant differences between the placebo and probiotic groups. Prematurity is another known factor that affects gut microbiota specifically by reducing bacterial diversity and is enriched in pathogenic taxa [104,105]. Although the composition of gut microbiota in preterm children becomes more similar to that of term children as they grow, subtle differences persist [106,107]. Recent studies found that gestational age has an impact on the severity and duration of gut dysbiosis, whereby the more premature the infant, especially <32 weeks of gestation, the deeper and longer lasting the gut dysbiosis [107,108,109]. Thus, excluding children with a history of extreme or very preterm birth would help strengthen the validity of the study.

Apart from that, breastfeeding alters the gut microbiota strongly in early life, and these compositional and functional signatures persist at least through the second year and sometimes beyond, with the duration and exclusivity of breastfeeding modulating the magnitude of long-term effects [110,111]. This makes controlling breastfeeding as a confounding factor important for validating the effect of probiotics in ASD children. Medication use, such as antibiotics, NSAIDs, statins, opioids, psychiatric medications, and PPIs, was found to affect gut microbiota composition and diversity [112,113,114].

### 4.8. Role of Vitamin D Status on Efficacy of Probiotics

One of the studies had reported that children who took probiotics with ADOS score improvement had significantly higher baseline 25(OH)D status than those who did not have an ADOS score improvement [45]. This finding shows there is a possible synergistic effect of baseline 25(OH)D status with the efficacy of probiotics in improving core ASD symptoms. Human studies consistently found that vitamin D supplementation significantly increased gut microbial diversity [115,116]. In children, vitamin D inadequacy was associated with reduced diversity, higher Firmicutes/Bacteroidetes ratio, and Prevotella-dominated enterotypes, giving an unfavorable configuration [117]. Vitamin D generally increases Bifidobacterium, Lactobacillus, Akkermansia, Faecalibacterium, and other health-associated taxa in gut microbiota while reducing Firmicutes and potential pathogens [115,116]. Vitamin D was thought to improve gut microbiota composition by regulating antimicrobial peptides, tight junction proteins, and mucosal immunity via the vitamin D receptor (VDR) in intestinal epithelium and immune cells [118,119,120]. Many guidelines converge on 400–800 IU/day of vitamin D supplementation for children as a safe preventive range, with targets of serum 25(OH)D ≥ 20 ng/mL [121,122,123].

### 4.9. Probiotics as a Nutritional Intervention

From a nutritional perspective, probiotics should be considered structured dietary interventions rather than solely adjunctive therapeutic agents. As live microorganisms administered to modulate gut microbiota composition and metabolic activity, their effects may be influenced by baseline dietary patterns and micronutrient status. Combined probiotic and nutritional interventions have been demonstrated to be beneficial across various patient populations, including lung cancer chemotherapy patients [124], malnourished hemodialysis patients [125], and stroke patients [126]. The possible synergistic effects of probiotic and nutritional intervention in ASD patients could be examined in the future. Therefore, future nutrition-controlled trials should incorporate standardized dietary assessments, control for macro- and micronutrient intake, and stratify participants according to baseline gut-related symptoms to minimize confounding.

### 4.10. Strengths and Limitations

This systematic review provides a detailed overview of the current evidence on the clinical efficacy of probiotics in improving core ASD symptoms in pediatric populations. A key strength of this review is the analysis of outcomes according to specific domains of core ASD symptoms, an approach that remains relatively underexplored and may help guide more targeted future research. In addition, we conducted a comprehensive appraisal of methodological quality and outcome evaluation tools used across existing studies, highlighting important sources of heterogeneity and bias. This analysis may serve as a foundation for future trials to adopt more standardized and rigorous methodologies to generate higher-quality evidence in this field. Finally, by examining probiotic strains, dosages, and intervention protocols, this review offers a thorough picture of current research practices and identifies areas requiring greater consistency and refinement. This systematic review is subject to several common limitations. The substantial heterogeneity across included studies in terms of design, participant characteristics, interventions, outcome measures, and follow-up duration limited direct comparison and made a meta-analysis not feasible. This systematic review was not prospectively registered in PROSPERO. Variability in methodological quality, including small sample sizes, inadequate blinding, and attrition, may have influenced the reliability of the reported effects. The use of diverse outcome assessment tools, particularly a mix of objective clinician-rated and subjective caregiver-reported measures, further complicated interpretation. In addition, publication bias cannot be excluded, as studies with positive findings are more likely to be published, and short follow-up periods restrict an evaluation of long-term treatment effects.

## 5. Conclusions

Generally, most studies reported a variable degree of improvement of core ASD symptoms after probiotic intervention, with a focus on the social and communication domain. However, only a limited number of high-quality studies demonstrated benefits. The evidence on improving other domains, such as restrictive and repetitive behaviors of core ASD symptoms, was limited and weak. The most common evaluation tools used to assess the core ASD symptoms were SRS, ATEC, and ADOS. Current evidence suggests *L. reuteri* supplementation for at least three months possibly has a role in improving the social and communication function of ASD children. However, further studies were needed to prove this hypothesis. Interventions with other probiotic strains gave inconsistent results, and the dosage of probiotics was greatly variable across studies.

This systematic review provides a novel insight into the efficacy of probiotics across different domains of core ASD symptoms. The effect of probiotics on core ASD symptoms was found to be strain-specific and domain-specific, with a low amount of certainty. Considerable amounts of studies have a high risk of bias, which further weakens the strength of evidence. Future studies should examine more of *L. reuteri*’s potential in ameliorating social and communication deficits in ASD children, using ADOS as the evaluation tool if resources permit. Moreover, probiotics should be considered as a nutritional intervention, while controlling confounding factors such as diet, nutritional status, and concurrent interventions should be controlled as much as possible to strengthen internal validity. More multi-centered and well-designed studies with standardized objective scoring scales, strain-specific hypotheses, and domain-based discussion are needed to gain more high-quality and comprehensive evidence on the clinical efficacy of probiotics in the treatment of ASD children.

## Figures and Tables

**Table 2:** Results of methodological quality assessment of nonrandomized studies.

First Author, Year	Risk Due to Confounding	Risk in Classification of Interventions	Risk in Selection of Participants into the Study	Risk Due to Deviations from Intended Interventions	Risk Due to Missing Data	Risk Arising from Measurement of the Outcome	Risk in Selection of the Report Result	Overall Risk of Bias
Mensi, 2021 [47]	-	-	-	-	-	-	-	Critical (not to proceed with assessment)
Li, 2024 [31]	Low	Low	Low	Low	Serious	Serious	Low	Serious
Niu, 2019 [36]	Serious	Low	Low	Low	Low	Moderate	Serious	Serious
Shaaban, 2017 [42]	Serious	Moderate	Low	Low	Low	Moderate	Low	Serious

**Table 4:** Summary table of the study’s risk of bias against the reported main findings.

Type of Study	Risk of Bias	Domains Reported to Have Significant Improvement After Probiotic Intervention				Improvement in Overall Score	Related to Metabolite Change	No Significant Improvement
		SC	RRB	AF/DLS	SCP			
RCTs	Low	Mazzone et al. [37]	-	-	-	-	-	Liu et al. [29] Rojo-Marticella et al. [49]
	Some concerns	Khanna et al. [30]	Khanna et al. [30]	-	-	-	-	Arnold et al. [43] Billeci et al. [46]
	High	Guidetti et al. [38] Santocchi et al. [44]	-	Santocchi et al. [44]	-	-	Guiducci et al. [45] Sherman et al. [48]	-
Non-RCTs	Serious	Niu et al. [36] Shaaban et al. [42]	Niu et al. [36] Shaaban et al. [42]	Niu et al. [36] Shaaban et al. [42]	Niu et al. [36] Shaaban et al. [42]	Li et al. [31]	-	-
	Critical	-	-	-	-	Mensi et al. [47]	-	-

Abbreviations: AF/DLS: adaptive functioning/daily living skills; RRB: repetitive and restrictive behavior; RCT: randomized-controlled trial; SC: social and communication; SCP: sensory and cognitive processing.

**Table 5:** Summary of the ASD evaluation tool used and the results reported.

Mode of Evaluation	ASD Evaluation Tool	Author	Risk of Bias	Significant Improvement in Tool Measured	Percentage of Significant Improvement Reported
Objective evaluation by clinician	ADOS/ADOS-2	Mazzone et al. [37]	Low	No	50.00%
		Billeci et al. [46]	Some concerns	No	
		Santocchi et al. [44]	High	Yes (Only in NGI group)	
		Guiducci et al. [45]	High	Yes (Indirect)	
	CARS	Billeci et al. [46]	Some concerns	No	
		Li et al. [31]	Serious	Yes	
Mixed objective evaluation by clinician and subjective reporting by caretaker	VABS/VABS-II	Billeci et al. [46]	Some concerns	No	75.00%
		Santocchi et al. [44]	High	Yes (Only in GI group)	
		Guidetti et al. [38]	High	Yes	
	ABAS-2	Mazzone et al. [37]	Low	Yes	
Subjective reporting by caretaker	ATEC	Niu et al. [36]	Serious	Yes	58.33%
		Shaaban et al. [42]	Serious	Yes	
	ABC/ABC-2	Liu et al. [29]	Low	No	
		Khanna et al. [30]	Some concerns	Yes	
		Arnold et al. [43]	Some concerns	No	
		Sherman et al. [48]	High	Yes (Indirectly)	
	SRS/SRS-2	Mazzone et al. [37]	Low	Yes	
		Li et al. [31]	Low	No	
		Rojo-Marticella et al. [49]	Low	No	
		Khanna et al. [30]	Some concerns	Yes	
		Arnold et al. [43]	Some concerns	No	
		Sherman et al. [48]	High	Yes (Indirectly)	

Abbreviations: ABAS-2: Adaptive Behavior Assessment System—Second Edition; ABC: Aberrant Behavior Checklist; ABC-2: Aberrant Behavior Checklist—Second Edition; ADOS: Autism Diagnostic Observation Schedule; ADOS-2: Autism Diagnostic Observation Schedule, Second Edition; ATEC: Autism Treatment Evaluation Checklist; SRS: Social Responsiveness Scale; SRS-2: Social Responsiveness Scale, Second Edition; VABS: Vineland Adaptive Behavior Scales; VABS-II: Vineland Adaptive Behavior Scales—Second Edition.

## Data Availability

The original data presented in the study are openly available in FigShare at doi:10.6084/m9.figshare.31074418.

## Supplementary Materials

The following supporting information can be downloaded at: https://www.mdpi.com/article/10.3390/nu18071127/s1. PDF S1: PRISMA 2020 Checklist. PDF S2: Research database. Reference [127] is cited in the supplementary materials.

## Abbreviations

The following abbreviations are used in this manuscript:

ASD	Autism spectrum disorder
ADOS	Autism Diagnostic Observation Schedule
ADOS-2	Autism Diagnostic Observation Schedule—Second Edition
CARS-2	Childhood Autism Rating Scale—Second Edition
ABC	Autism Behavior Checklist
ATEC	Autism Treatment Evaluation Checklist
VABS-II	Vineland Adaptive Behavior Scales, Second Edition
SRS	Social Responsiveness Scale
AS	Asperger Syndrome
PDD-NOS	Pervasive Developmental Disorder Not Otherwise Specified
FMT	Fecal microbiota transplantation
PRISMA	Preferred Reporting Items for Systematic Reviews and Meta-Analyses
RoB 2	Version 2 of the Cochrane risk-of-bias tool for randomized trials
ROBINS-I V2	Risk Of Bias in Non-randomized Studies—of Interventions, Version 2
ABAS-2	Adaptive Behavior Assessment System—Second Edition
ABC-2	Aberrant Behavior Checklist—Second Edition
ABC-T	Autism Behavior Checklist—Taiwan Version
ADOS-2	Autism Diagnostic Observation Schedule, Second Edition
ADOS-CSS	Autism Diagnostic Observation Schedule—Calibrated Severity Score
ASRS	Autism Spectrum Rating Scales
CBCL	Child Behavior Checklist
CFU	Colony-forming units
CGI	Clinical Global Impressions Scale
CPT-3	Conners Continuous Performance Test—Third Edition
EEG	Electroencephalogram
K-CPT 2	Kiddie Conners Continuous Performance Test—Second Edition
LP	*Lactobacillus plantarum*
OP	Other probiotic
PEP	Psychoeducational Profile
PGIA	Parent Global Impression of Autism
PLA	Placebo
PRO	Probiotic
RBS-R	Repetitive Behavior Scale—Revised
RRB	Restricted and repetitive behaviors
SCQ	Social Communication Questionnaire
SNAP-IV	Swanson, Nolan, and Pelham Rating Scale—Version IV
SRS-2	Social Responsiveness Scale—Second Edition
VABS	Vineland Adaptive Behavior Scales
GI	Gastrointestinal
ABA	Applied behavioral analysis
RCT	Randomized controlled trial
AF/DLS	Adaptive functioning/daily living skills
SC	Social and communication
SCP	Sensory and cognitive processing
